# Supplementary material for: An Artificial Intelligence-Enabled ECG Algorithm for the Prediction and Localization of Angiography-Proven Coronary Artery Disease
Source: Biomedicines. 2022 Feb 7;10(2):394. doi: 10.3390/biomedicines10020394 (PMC8962407; doi:10.3390/biomedicines10020394)
Supplement: Supplementary file 1 [file biomedicines-10-00394-s001.zip › Supplement Figure Legend Rev2.pdf]

Supplement Figure

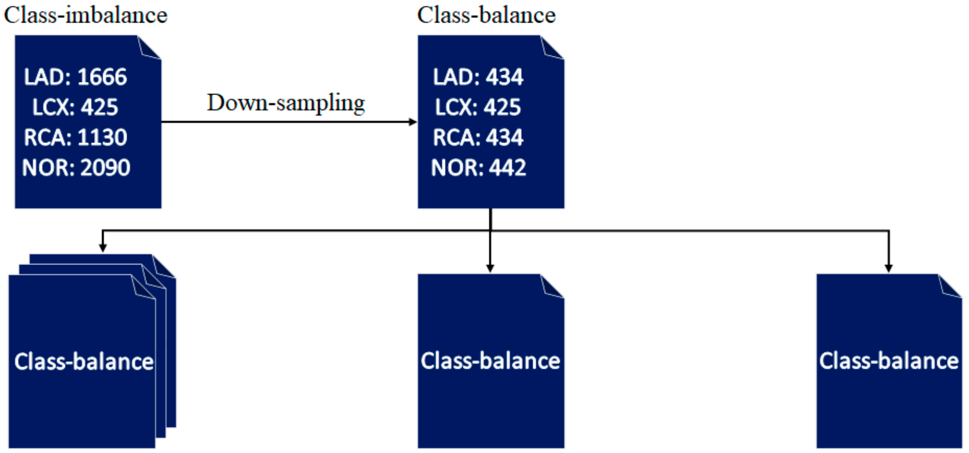

(a) Random selection (3 times)   (b) Subgroup 1 – with AMI & Ischemia   (c) Subgroup 2 – without AMI & Ischemia

※ LAD = left anterior descending artery, LCX = left circumflex artery, RCA = right coronary artery, NOR: normal, AMI = acute myocardial infarction.

(a) Random selection - During the down-sampling process, we randomly selected data in both LAD and RCA for 434 ECG records and NOR for 442 records to balance the data in each category. To ensure the generalization, we did this process three times and generated three random selection subsets.

(b) Subgroup 1 – By analyzed the random selection subset's results, we found that the subset with more AMI and ischemia symptom's ECG record would have better performance. We assumed the ECG with the symptom of AMI and ischemia could provide more discriminated features to make CNN model classify better. To verify our hypothesis, we intentionally selected only ECGs with AMI and ischemia symptoms to form a subset (called subgroup 1) at the time of down-sampling. As long as it got better performance than random selection subset, our hypothesis should be validated.

(c) Subgroup 2 – In contrast, we intentionally selected only ECGs without any AMI and ischemia symptoms to form a subset (called subgroup 2) at the time of down-sampling. To observe how much of an effect there is when the model loses learning features from ECGs with AMI and ischemic symptoms. If poorer performance is obtained then verify the symptoms of AMI and ischemia are crucial features to locate obstruction of the coronary artery.

Supplement Figure S1. Class-balance subsets selected criteria.
(a) Random selection - During the down-sampling process, we randomly selected data in both LAD and RCA for 434 ECG records to balance the data in each category. To ensure the generalization, we did this process three times and generated three random selection subsets. (b) Subgroup 1 – By analyzed the random selection subset's results, we found that the subset with more AMI and ischemia symptom's ECG record would have better performance. We assumed the ECG with the symptom of AMI and ischemia could provide more discriminated features to make CNN model classify better. To verify our hypothesis, we intentionally selected only ECGs with AMI and ischemia symptoms to form a subset (called subgroup 1) at the time of down-sampling. As long as it got better performance than random selection subset, our hypothesis should be validated.
(c) Subgroup 2 – In contrast, we intentionally selected only ECGs without any AMI and ischemia symptoms to form a subset (called subgroup 2) at the time of down-sampling. To observe how much of an effect there is when the model loses learning features from ECGs with AMI and ischemic symptoms. If poorer performance is obtained then verify the symptoms of AMI and ischemia are crucial features to locate obstruction of the coronary artery.

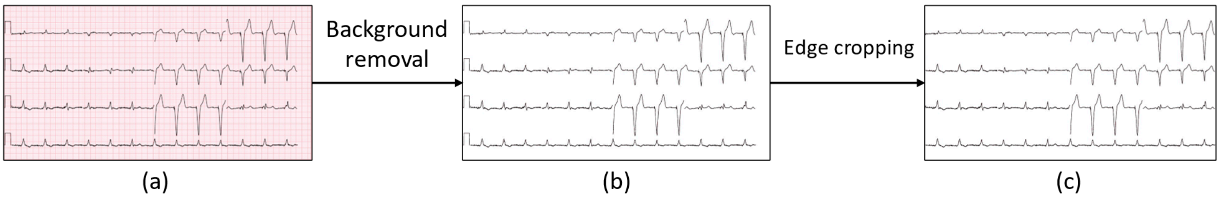

Supplement Figure S2. Data preprocessing.
(a) Standard 12-lead ECG image.
(b) Background removal 12-lead ECG image.
(c) Cropped 12-lead ECG image.
