# Supplementary material for: An Artificial Intelligence-Enabled ECG Algorithm for the Prediction and Localization of Angiography-Proven Coronary Artery Disease
Source: Biomedicines. 2022 Feb 7;10(2):394. doi: 10.3390/biomedicines10020394 (PMC8962407; doi:10.3390/biomedicines10020394)
Supplement: Supplementary file 1 [file biomedicines-10-00394-s001.zip › Supplement Table Rev2.pdf]

Supplement Table S1. Application programming interfaces (API)s and parameter settings.

Hyperparameter	Image input model	Time-series input model
Convolutional kernel size	Set by default	[3, 5, 7, 9, 11, 13, 15, 17, 19,21]
Epoch	200	400
Batch size	32	32
EarlyStopping patience	100	200
Dropout rate	0.5	0.5
Optimizer	Adam	Adam
Learning rate	10-3	10-3
Metrics	Accuracy	Accuracy
Loss function	Categorical crossentropy	Categorical crossentropy

Supplement Table S2. Randomly selected subsets test results.

Image input model

Subset	Acc.	AUC				Precision	Recall
		NOR	LAD	LCX	RCA		
1	0.698 ±0.01	1.0 ±0.0	0.892 ±0.017	0.764 ±0.027	0.796 ±0.018	0.699 ±0.016	0.696 ±0.008
2	0.7 ±0.018	1.0 ±0.0	0.850 ±0.014	0.782 ±0.019	0.824 ±0.033	0.705 ±0.015	0.698 ±0.015
3	0.738 ±0.007	1.0 ±0.0	0.914 ±0.012	0.782 ±0.022	0.828 ±0.029	0.743 ±0.005	0.736 ±0.006
AVG	0.712 ±0.012	1.0 ±0.0	0.885 ±0.016	0.776 ±0.013	0.816 ±0.016	0.716 ±0.012	0.71 ±0.011

AUC: The area under curve, LAD: Left anterior descending, LCX: Left circumflex, RCA: Right coronary artery, AVG: Average.
